# RAB8A a new biomarker for endometrial cancer?

**DOI:** 10.1186/1477-7819-12-371

**Published:** 2014-12-04

**Authors:** Yachun Bie, Zhenyu Zhang

**Affiliations:** Department of Obstetrics and Gynecology, Beijing Chao-yang Hospital affiliated to Capital Medical University, No. 8 Gongti South Road, Beijing, 100020 China

**Keywords:** Proteomic analysis, Dendritic cells (DCs), Endometrial cancer (EC), Tumor lysate, LC-MS/MS, Biomarker

## Abstract

**Background:**

We aimed to find different proteins between dendritic cells (DCs) and tumor antigen-pulsed DCs to help find a new biomarker for endometrial cancer (EC).

**Methods:**

Mononuclear cells were isolated from cord blood and induced to DCs by cytokines. A comparative proteomic analysis was performed on DCs and tumor lysate-pulsed DCs by liquid chromatography with tandem mass spectrometry (LC-MS/MS). Differential proteins were identified by Western blot analysis.

**Results:**

The expression of *Ras-related protein Rab-8A* (*RAB8A*) was found to be different in the in two kinds of cells. This phenomenon was also proven in endometrial cancer tissues.

**Conclusions:**

*RAB8A* might be a new biomarker for endometrial cancer. Using LC-MS/MS to perform a comparative proteomic analysis about DCs and tumor lysate-pulsed DCs may help us to find new biomarker of cancer.

## Background

Endometrial cancer (EC) is a common gynecological malignancy and the incidence is gradually increasing due to life patterns [1]. Current methods of diagnosis of EC rely on invasive techniques, such as biopsy and curettage. There are no specific tumor markers for EC. Identification of tumor antigens is important not only for studying antitumor immune responses and immunotherapy, but also for the development of diagnostic methods [2].

It is known that dendritic cells (DCs) are the most potent antigen-presenting cells and can be derived *ex vivo* from monocytes of blood by using GM-CSF (granulocyte-macrophage colony-stimulating factor) and IL-4 (Human interleukin 4) [3]. Immunotherapy with DCs has been studied in a wide variety of cancers and has demonstrated the induction of tumor-specific immune responses. Therefore, we assumed that protein expressions of DCs are different before and after pulsing with tumor lysate. The results could be useful in helping to find tumor-specific antigens of EC between these differences. We performed a comparative proteomic analysis of DCs and tumor lysate-pulsed DCs by liquid chromatography with tandem mass spectrometry (LC-MS/MS). We used the HEC-1-B cell line of EC to prepare the tumor lysate.

## Methods

Informed consent was obtained from all the patients before enrollment in the study. This study was approved by the institutional Ethics Committees of Beijing Chao-yang Hospital affiliated to Capital Medical University and conducted in accordance with the ethical guidelines of the Declaration of Helsinki.

### Cell line culture medium and tumor lysate preparation

HEC-1-B cells were cultured in DMEM/F12 (dulbecco's modified eagle medium) (HyClone, Waltham, Massachusetts, United States) medium supplemented with penicillin (10 IU/ml), streptomycin (100 ug/ml), and 10% fetal bovine serum (FBS, Gibco, Carlsbad, California, United States), and in a humidified atmosphere of 5% CO2 at 37°C. They were harvested when there was 80 to 90% confluent and rinsed twice with PBS. 1 × 10^7^/ml cells were lysed by five freeze cycles in liquid nitrogen and thaw cycles at room temperature. They were then centrifuged at 400 g for 10 minutes, then supernatants were passed through a 0.2 um filter (PAll Acrodisc® United States) and stored at -80°C [4].

### Isolation of umbilical cord blood mononuclear cells and generation of dendritic cells

Umbilical cord blood samples were collected from normal full-term deliveries after obtaining written informed consent. Umbilical cord blood mononuclear cells (UBMCs) were separated from 50 ml fresh cord blood with heparin (200 IU/ml) by Ficoll-Hypaque 1.077 g/ml (Gibco-Invitrogen, Paisley, United Kingdom), subjected to density gradient centrifugation and placed into six-well culture plates in RPMI (Roswell Park Memorial Institute) 1640 medium plus 10% FBS (Gibco-BRL Gaithersburg, Maryland, United States) at 1 × 10^6^/2 ml per well. After two hours at 37°C in a humidified 5% CO_2_ incubator, nonadherent cells were removed, and the adherent cells were cultured in same medium supplemented with recombinant human GM-CSF (1,000 U/ml) and IL-4 (1,000 U/ml) (PeproTech, United States). Every three days, 1 ml of spent medium was replaced by 2 ml of fresh medium containing GM-CSF and IL-4, to yield final concentrations of 1,000 U/ml.

### Dendritic cell pulsing

After five days of culture, immature DCs were harvested and suspended in medium with GM-CSF and IL-4 at 1 × 10^6^/ml and mixed with tumor lysate at ratios of 1:10. DCs with or without antigen loading were harvested on the seventh day.

### Flow-based analysis of labeled cells

Cells were washed with PBS twice and incubated in a 10% FcR-blocking solution (Miltenyi Biotec Bergisch Gladbach, Germany) for 30 minutes at 4°C to block the nonspecific binding to FcR. Then, cells were stained with PE- or APC-conjugated antibodies. Control staining was performed simultaneously using isotype-matched, irrelevant antibodies also directly conjugated to PE or APC. The fluorescence intensity of the cells was analyzed by flow cytometry (FACS Calibur; Becton Dickinson, United States). The antibodies used are PE-conjugated anti-CD80 and HLA-DR (BD Biosciences, United States) and APC-conjugated anti-CD11c (BD Biosciences, United States).

### Sample preparation for LC-MS/MS

Cells, including DCs and DCs with HEC-1-B cells lysate-pulsed, were washed twice with ice-cold PBS containing protease inhibitors and sonicate in ice-cold RIPA buffer (Sangon Biotech, Shanghai, China) and centrifuged at 1,000g at 4°C for 10 minutes. The supernatant proteins were boiled for five minutes before analysis on 12% polyacrylamide gels (Sangon Biotech, Shanghai, China). Protein concentrations were determined with the bicinchoninic acid (BCA) method (Thermo Scientific, United States). Gels were placed in a staining solution (1.3 M ammonium sulfate, 34% methanol, 1.4% orthophosphoric acid and 0.07% Coomassie Brilliant Blue G250 (Sangon Biotech, Shanghai, China) for 24 hours, and then were de-stained until the background was clear. Coomassie Blue-stained protein bands were excised from the SDS-PAGE gel. Gel pieces were de-stained twice for 10 minutes in a solution containing 100 mM NH4HCO_3_ and 50% acetonitrile, dehydrated in acetonitrile, and dried in a vacuum centrifuge. Then, gel pieces were rehydrated at 4°C for 45 minutes in a digestion buffer (50 mM NH4HCO3 and 12.5 ng/ul trypsin). The supernatant was replaced by 40 ul of 50 mM NH4HCO_3_ and the samples were incubated overnight at 37°C. The tryptic peptides were recovered by 10-minute incubations, once in 25 mM NH4HCO_3_ and 50% acetonitrile, and twice in 25 mM NH4HCO_3_, 50% acetonitrile and 5% formic acid (Sangon Biotech, Shanghai, China). All supernatants were pooled and dried in a vacuum centrifuge [5].

The resulting peptides were analyzed by LC-MS/MS using a capillary LC system (Magic2002; Michrom BioResources, Auburn, California, United States) coupled to an inline nano-electrospray mass spectrometer (LCQ Advantage; Thermo Finnegan, Waltham, Massachusetts, United States) with a silica-coated glass capillary tube (PiclTip; New Objective, Woburn, Massachusetts, United States). Raw LC-MS/MS data were analyzed by searching the Mascot database [6] to identify the proteins. To generate a statistically valid list of proteins, Scaffold, United States was used to accommodate differences of algorithm and score calculation by the two search engines [7].

### Western blotting

Western blotting analysis was used to validate the findings. Total proteins were extracted using a RIPA kit (Beyotime, Shanghai, China) containing a 1% dilution of the protease inhibitor PMSF (Phenylmethanesulfonyl fluoride) (Beyotime, Shanghai, China). Protein concentrations were determined with the BCA method. Proteins (50 ug/lane) were separated by 12% SDS-PAGE and transferred to a 0.2 um PVDF (Polyvinylidene Fluoride, Beyotime, Shanghai, China) membrane (Millipore, Billerica, Massachusetts, United States). After blocking the membrane in blocking buffer (5% milk powder in 20 mM Tris-HCl pH 7.5, 500 mM NaCl, 0.1% (v/v) Tween 20 (Beyotime, Shanghai, China), the membrane was incubated with primary antibodies against *RAB8A* (1:1,000; Proteintech, United States) and tubulin (1:2,000, Cell Signaling Technology, United States) at 4°C overnight. Peroxidase-linked secondary anti-rabbit or anti-mouse antibodies were used to detect the bound primary antibodies. Enhanced chemiluminescence (ECL) reagents (Santa Cruz) were used to visualize (Bio-rad, United States).

## Results

**Figure 1:**
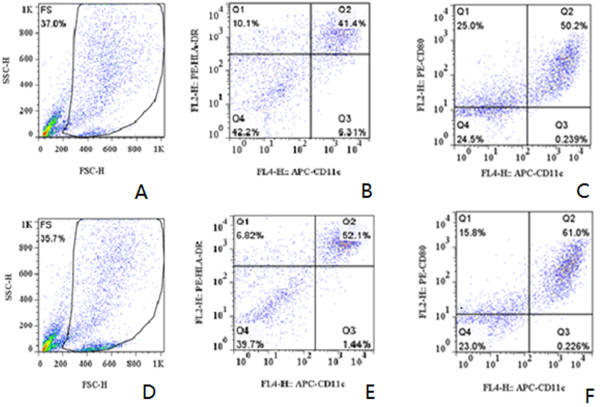
**Flow cytometric analysis of the seventh day DCs. A**-**C** show the cells were cultured by the adherent method, and **D**-**F** show that the DCs were pulsed with HEC-1-B cell lysate. The cells were harvested at the seventh day of culture. The cells in the gated population were further analyzed and found to be CD11C^+^/HLA-DR^+^ **(B, E)** and CD11C^+^/CD80^+^ **(C, F)**. DCs dendritic cells.

Figure 1 is flow cytometric analysis result. Figure 1A to C show that the DCs were cultured using UBMC by the adherent method and Figure 1D to F show that the DCs were pulsed with HEC-1-B cell lysate. The cells were harvested at the seventh day of culture. The cells in the gated population were further analyzed and found to be CD11C^+^/HLA-DR^+^ (Figure 1B and E) and CD11C^+^/CD80^+^ (Figure 1C and F). From the flow cytometric analysis we can conclude that the tumor cell lysate of EC can mature DCs.

**Figure 2:**
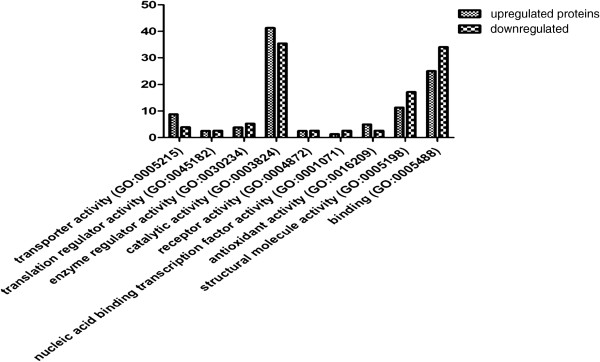
**The comparison of protein function between upregulated and downregulated proteins.** Functions of transporter activity, catalytic activity and antioxidant activity are significantly higher in upregulated proteins (*P* <0.05).

**Figure 3:**
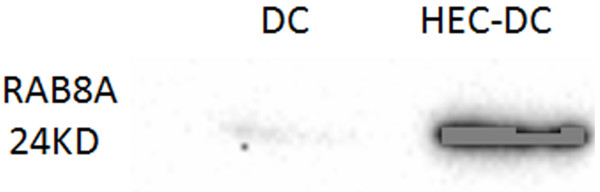
**The expression of** ***RAB8A*** **in cells.** The expression of *RAB8A* is significantly higher in HEC-DC than in DC and HEC-1-B cells as analyzed by Western-blot (*P* <0.05). DCs dendritic cells

**Figure 4:**
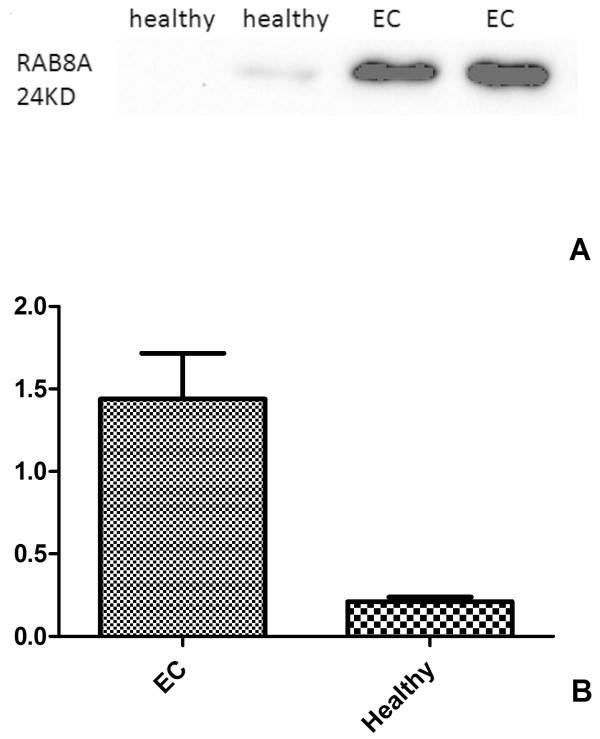
**The expression of** ***RAB8A*** **in healthy and malignant endometrium tissues (A, B).** A significant increase of *RAB8A* in EC versus the healthy controls was observed by Western blot analysis (*P* <0.05). **(A)** the image of Western-blot in EC and healthy tissues; **(B)** Analysis of the Western-blot image results in EC and healthy tissues. EC endometrial cancer.

Protein expression of cells, including DCs and DCs with HEC-1-B cell lysate pulsed (HEC-DCs), were analyzed by LC-MS/MS. We found 136 upregulated proteins and 110 downregulated proteins in HEC-DCs compared with DCs. Figure 2 shows the comparison of protein function between upregulated and downregulated proteins. Some proteins that were upregulated were chosen to be analyzed by Western blotting. We found that the expression of *Ras-related protein Rab-8A (RAB8A)* is significantly higher in HEC-DC than in DCs (Figure 3). We also compared its expression in tissues of healthy and malignant endometrium. A significant increase of *RAB8A* in EC versus healthy controls was observed (*P* <0.05) (Figure 4).

## Discussion

In the early 1970s, DCs were first identified [8]. Now it is widely recognized that DCs are the major antigen-presenting cells (APCs) to T-lymphocytes, which not only initiate, but also control cellular immune responses [9]. Most published research show results from experiments using magnetic activated cell sorting (MACS) separation CD34^+^ cells from cord blood to induce DCs. In this study, we also induce DCs from cord blood using the adherent method; proven by flow cytometric analysis. We also show that the HEC-1-B tumor cell lysate can mature DCs.

From the comparative proteomic analysis by LC-MS/MS, functions of transporter activity, catalytic activity and antioxidant activity are higher in upregulated proteins. Some proteins, such as *Vimentin (VIM), Rho GDP-dissociation inhibitor 1 (Rho GDI), Lysosome-associated membrane glycoprotein 1 (LAMP1)* and *Membrane-associated progesterone receptor component 2 (PGRMC 2)*, have been previously identified as potential biomarkers for various cancers [10–14]. By reviewing articles, only one of them has been studied in EC [15].

In our study, we found a significant increase of *RAB8A* in EC, which is a small GTPase belonging to the rab family of ras-GTPases, and is also a marker of recycling endosomes [16]. *RabGTPases* are major regulators of intracellular trafficking that act as molecular switches, with their on/off regulatory function considered to be restricted to the membrane compartments where they are located [17]. Alterations in *Rab-GTPase* expression or activity can cause defects in cell adhesion, motility and invasion, leading to neurologic diseases, lipid storage disorders or cancer [18]. Recently, *RAB8A* was found to control cell surface exposure of *MT1-MMP* (membrane type-1 matrix metalloproteinase), extracellular matrix degradation and three-dimensional invasion of macrophages [17]. *MT1-MMP* is thought to play a key role in tumor invasion. The highest expression of *RAB8* was at an early stage of cisplatin resistance. *RAB8* and TMEM205 indicated an additive effect of mediating cisplatin resistance [19].

Sun *et al*. [20] concluded that *RAB13* and *RAB8A* are Rab-GTPases activated by insulin, and that downstream of *AS160* they regulate traffic of *GLUT4(Glucose transporter 4)* vesicles, possibly acting at distinct steps and sites. These findings close in on the series of events regulating muscle *GLUT4* traffic in response to insulin, crucial for whole-body glucose homeostasis [20]. Our research may help to explain the cause of EC in patients with obesity and diabetes.

## Conclusions

The adherent method can be used in cord blood to induce DCs. As DCs are the most potent antigen-presenting cells, we used this character to perform a comparative proteomic analysis of DCs and tumor lysate-pulsed DCs by LC-MS/MS, and found the expression of *RAB8A* to be significantly higher in HEC-DC and malignant endometrium tissues. We hypothesize that *RAB8A* may participate in the progression of malignant growth of EC. However, it is still too early to conclude *RAB8A* as a novel biomarker for EC diagnosis. The role of *RAB8A* in EC needs to be further studied. We hope our results will provide useful information for studies on the detection and therapy of EC.

## Abbreviations

DC: dendritic cell
EC: endometrial cancer
GM-CSF: granulocyte-macrophage colony-stimulating factor
GLUT4: Glucose transporter 4
IL-4: Human interleukin 4
MASC: Immune magnetic bead separation of cells
MT1-MMP: membrane type-1 matrix metalloproteinase
PMSF: Phenylmethanesulfonyl fluoride
PVDF: Polyvinylidene Fluoride
UBMC: Umbilical cord blood mononuclear cell.
